# A Mobile Gaming App to Train Teenage Mothers on Appropriate Child Feeding Practices: Development and Validation Study

**DOI:** 10.2196/53560

**Published:** 2024-09-26

**Authors:** Mercy Eloho Sosanya, Folake Olukemi Samuel, Sadia Bashir, Victoria Osariemen Omoera, Jeanne H Freeland-Graves

**Affiliations:** 1 Department of Nutritional Sciences University of Texas at Austin Austin, TX United States; 2 Department of Nutrition and Dietetics The Federal Polytechnic, Bauchi Bauchi Nigeria; 3 Department of Human Nutrition and Dietetics College of Medicine University of Ibadan Ibadan Nigeria; 4 PixelArt Games Academy Islamabad Pakistan; 5 Centre for Family Health Initiative Abuja Nigeria

**Keywords:** mobile health, mHealth, mobile gaming app, validation, infant and young child feeding, teenage mother, Nigeria, mobile phone

## Abstract

**Background:**

Undernutrition is an underlying factor in nearly 50% of 1 million estimated annual deaths among Nigerian children aged <5 years. Inappropriate maternal infant and young child feeding (IYCF) practices are basic contributors to child undernutrition. Teenage motherhood exacerbates the problem of inadequate child feeding. One possible intervention method to improve IYCF knowledge and practices of teenage mothers is the use of mobile gaming technologies. Despite extreme poverty in low- and middle-income countries, a ubiquity of mobile phone networks exists.

**Objective:**

This study aims to develop and validate a mobile gaming app, called BabyThrive, to train Nigerian teenage mothers on appropriate IYCF practices.

**Methods:**

To identify gaps in current IYCF practices in northern Nigeria, we conducted an extensive search of the literature and held 2 focus group interviews with 16 teenage mothers with low-income status. An initial app content design was then created, and content validity was established by 10 nutrition experts. Next, we developed an app prototype, which was assessed for quality by 7 nutrition and mobile gaming experts and evaluated for usability by 90 teenage mothers from rural areas in Abuja, the country’s capital. The final app, BabyThrive, is a 2D mobile game that is fully functional offline and available in English as well as Hausa, which is commonly spoken in northern Nigeria. The efficacy of the BabyThrive app was assessed using IYCF knowledge scores obtained from the administration of the validated Teen Moms Child Feeding Questionnaire for Sub-Saharan Africa. Construct validity was established via crossover design by comparing the total IYCF knowledge scores of the teenage mothers obtained after a verbal training program and BabyThrive app use.

**Results:**

Large proportions of the study participants were married (53/90, 59%) and had no personal income (63/90, 70%). The mean quality rating for the BabyThrive app was 4.3 (SD 0.39) out of 5.0. High levels (>80%) of usability and user satisfaction were documented. Knowledge of exclusive breastfeeding (*P*<.001) and total knowledge scores (*P*=.002) were significantly higher in the BabyThrive group than in the verbal training group. The IYCF knowledge scores obtained from both groups showed coherence, with a statistically significant Spearman correlation coefficient of 0.50 (*P*<.001).

**Conclusions:**

This research developed and validated a novel, offline mobile gaming app. It will be an easy, effective, and acceptable method to disseminate critical knowledge on IYCF practices to teenage mothers in rural Nigeria.

## Introduction

### Background

Adolescent girls (aged 15-19 y) in low- and middle-income countries account for almost 10% of 227 million global pregnancies, resulting in 12 million births each year [1-3]. Globally, sub-Saharan Africa has the highest percentage (46%) of adolescent girls bearing children [4]. Nigeria contributes to this high prevalence of teenage motherhood, with 38.3% of girls aged ≤19 years in rural areas having children [5]. Teenage mothers are generally undernourished and less educated, which often results in severely underweight infants who experience malnutrition [6,7]. In Nigeria, the prevalence of malnutrition in children aged 0 to 5 years is substantial: 37% are diagnosed with stunting, 18% with wasting, and 29% with underweight [8]. Furthermore, undernutrition is an underlying factor in nearly 50% of 1 million estimated annual deaths in Nigerian children aged <5 years [9,10]. In southwestern Nigeria, the situation is even worse among children born to teenage mothers: 73.4% were diagnosed with wasting, stunting, or underweight [11]. In northern Nigeria, children born to teenage mothers were 3 times more likely to die within the first 5 years after birth compared with children born to older mothers [12].

One basic contributor to this child undernutrition is inappropriate maternal infant and young child feeding (IYCF) practices [13]. A multiplicity of interventions have attempted to improve IYCF practices [14], but the results have been dismal. Less than one-third of children (29%) are exclusively breastfed for 6 months, only 42% of newborns are breastfed within 1 hour of birth, and almost half (49%) receive prelacteal feeds [10]. Similarly, less than a quarter of children aged 6 to 23 months receive meals of adequate diversity (at least 5 food groups included), and only 42% are fed the appropriate number of times for their age [10]. Even fewer children (11%) aged 6 to 23 months receive the minimum acceptable diet (a composite indicator of minimum dietary diversity and minimum meal frequency) [10].

The problem of inadequate child feeding is exacerbated by teenage motherhood. Previous studies have shown unacceptably low rates of early initiation, duration, and exclusivity of breastfeeding in this age group [15]. In comparison with adult mothers, poor complementary feeding practices are more frequent. These practices include improper timing of the initiation of complementary foods, less responsive feeding styles, and feeding of diets low in protein and high in fat and sugars [16]. In Nigeria, a 15-year trend analysis has documented consistently lower rates of timely breastfeeding initiation (43.7% vs 55%) and exclusive breastfeeding (24.8% vs 28.6%) among adolescent versus adult mothers, as well as higher rates of the use of prelacteal feeds (59.2% vs 49.7%) [17].

In northern Nigeria, IYCF interventions have been implemented by a collaboration of state governments and international development partners. These behavioral change interventions have been delivered to mothers via group and individualized counseling at health facilities by health workers, volunteer-led community counseling for mothers, and community meetings for fathers and grandmothers [18]. A qualitative evaluation of these IYCF programs identified numerous problems, including heavy work burden for health workers due to personnel shortages, limited financial remuneration for community volunteers, time constraints for personnel and participants, and challenges with transportation [18]. All these issues are potent threats to the viability and effectiveness of IYCF programs [18].

One possible intervention method to improve IYCF knowledge and practices of teenage mothers is the use of mobile health (mHealth) for the achievement of health objectives [19]. mHealth interventions can target a wider client base, with decreased costs, time, need for space, and requirements for personnel training [20]. Despite extreme poverty in low- and middle-income countries, a ubiquity of mobile phone networks exists. In fact, this network surpasses other infrastructure, including paved roads and electricity [19]. Nigeria, with a population of approximately 213 million, has >190 million mobile phone lines and approximately 105 million monthly mobile internet lines [21-23]. In African countries, including Nigeria, mothers who participated in IYCF interventions delivered via mobile voice and SMS text messages were 1.2 to 2.6 times more likely to practice exclusive and timely initiation of breastfeeding [24-26].

### The Focus of This Research

This research focuses on mobile games as a means of a nutrition intervention. Mobile games can be effective because they have the capacity to modify the attitudes, beliefs, behaviors, knowledge, and skills of users pertaining to health while providing entertainment [27,28]. These video games are designed to entertain users while eliciting changes in behavior and are often referred to as serious games [29]. Serious mobile games are useful in surmounting health challenges such as limited access to health information and may transcend social barriers, including low literacy and gender biases [30]. These games have shown promising results in a variety of health applications, including the improvement of treatment compliance, the rehabilitation of individuals with disabilities, obesity management, the promotion of healthy lifestyles, training parents on vegetable feeding practices for toddlers, and the improvement of knowledge and intention regarding healthy behaviors among older children [29,31,32]. Although gamification has been extensively used in numerous exercise and fitness apps, few apps have focused on changing maternal IYCF behaviors using this technique [33]. To date, no studies have explored the application of mobile gaming techniques to facilitate IYCF behavior change in Africa. Therefore, this study developed and validated a mobile gaming app, called BabyThrive, to train teenage mothers on appropriate IYCF practices.

## Methods

### App Development

**Figure 1:**
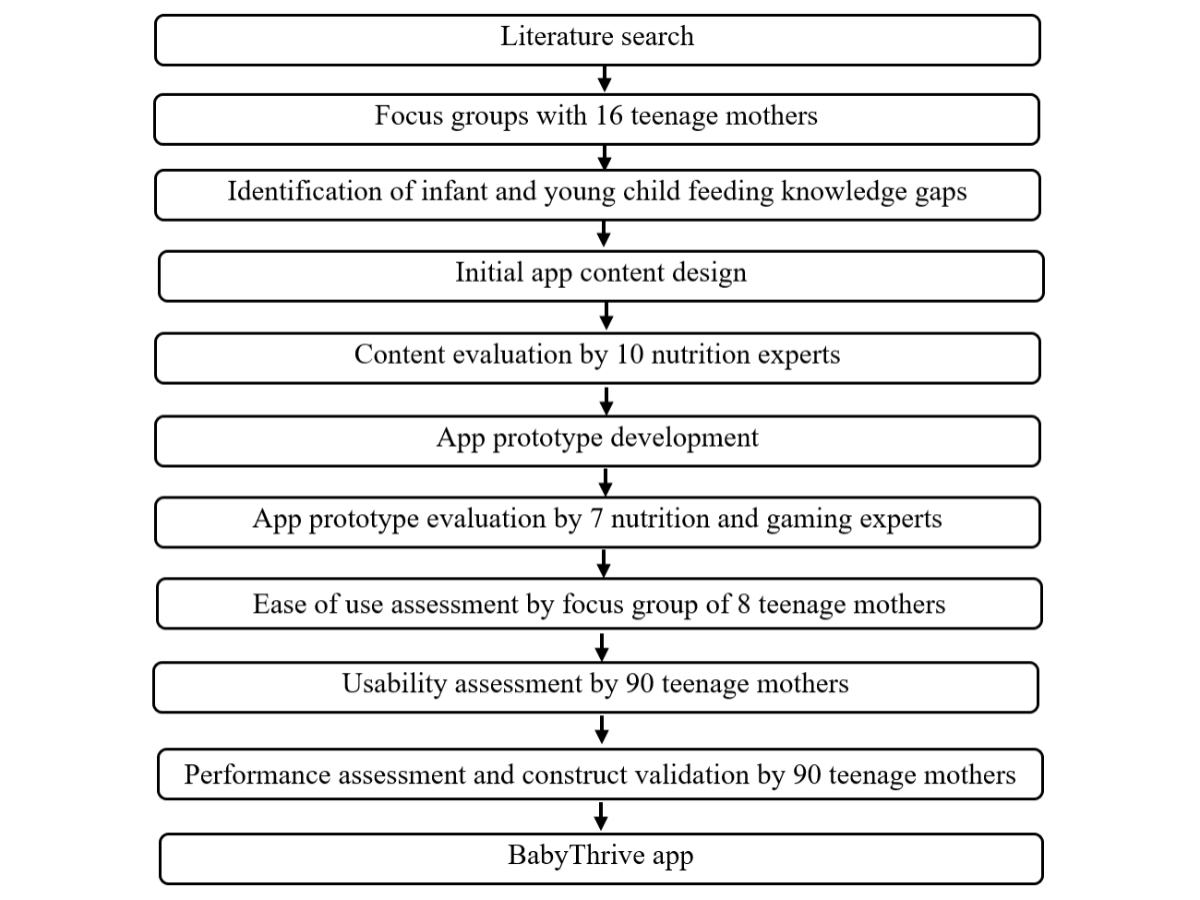
Flowchart for the development and validation of the BabyThrive app.

The mobile gaming app, BabyThrive, was developed through an iterative and incremental process. Figure 1 shows the flowchart for the development and validation of the app. We conducted an extensive literature search and held 2 focus group discussions with 16 teenage mothers with low-income status to identify gaps in current IYCF practices in northern Nigeria and effective behavior change techniques (BCTs) suitable for the study population. Guided by the findings from the literature search and focus group discussions, an initial app content design was created based on information adapted from the United Nations Children’s Fund (UNICEF) IYCF training package for Nigeria [34]. Permission to adapt content and images from the Community Infant and Young Child Feeding Key Messages Booklet (part of the training package) was obtained from the Federal Ministry of Health and Social Welfare in Abuja, the country’s capital.

The validity of the nutrition content of the initial app design was evaluated by a panel of 10 nutrition experts from family health, nutrition science, and dietetics departments at the University of Texas at Austin, Texas; the University of Ibadan, Ibadan, Nigeria; the Bauchi State Agricultural Development Program, Nigeria; and Nigeria’s Federal Ministry of Health in Abuja. Game art concepts, stories, dialogues, and user interface and user experience design were generated from the initial app content design, along with feedback from the panel of nutrition experts. These were incorporated into an app prototype developed using the Agile methodology [35] solely for the Android platform (the predominant mobile phone platform used in the study area) [36]. The app prototype underwent a series of in-house tests and correction of errors and then was evaluated for quality by 7 nutrition and mobile gaming experts using the Mobile App Rating Scale (MARS) [37]. These nutrition and gaming experts were recruited from the University of Illinois at Urbana-Champaign, Illinois; Federal Polytechnic Bauchi, Bauchi, Nigeria; Nigeria’s Federal Ministry of Health in Abuja; Kano State College of Health Sciences and Technology, Nigeria; Endoclan Applications (a game development firm), Port Harcourt, Nigeria; Abubakar Tafawa Balewa University, Bauchi, Nigeria; and GitLab Inc. Each quality component on the MARS was rated on a 5-point Likert scale ranging from 1=*inadequate* to 5=*excellent*, with a total possible score of 5. After modification based on suggestions from the experts, the app was assessed for comprehensibility and ease of use by a focus group of 8 teenage mothers in Nigeria. The feedback was integrated into the final version of the app.

The study has been registered with ClinicalTrials.gov (NCT05181293).

### App Description

#### Overview

The final app, BabyThrive, is a 2D character-animated mobile game featuring voice-acted audio dialogues. Multimedia Appendix 1 shows the app interface. The BabyThrive app is fully functional offline and provides 2 language options: English and Hausa (commonly spoken in northern Nigeria). The app contains 4 modules, which are described in the following subsections.

#### Interactive Game

This role-play video game with 5 levels, in which the player (a teenage mother) must learn, practice, and execute correct IYCF recommendations and decisions regarding her child’s health, provides detailed narratives concerning breastfeeding, including the benefits of exclusive breastfeeding to the child, mother, and community, as well as tips for successful breastfeeding, including techniques, position, attachment, the use of colostrum, concepts of foremilk and hindmilk, and breast milk expression. The game narratives and dialogues also cover complementary feeding concepts, including age-appropriate amounts, frequency and food group diversity, hygiene, responsiveness, and child feeding and care in illness. As young Nigerian mothers play the game, they identify with the game characters and interactively acquire knowledge to improve their practices and attitudes toward IYCF practices. To encourage active engagement in the learning process, BabyThrive uses simple gaming mechanics, including treasure hunts to find animated, informational cards; the dragging and dropping of matching objects; and the fixing of puzzles. These simple mechanics were used because evidence shows that extremely complex digital interventions are less effective [38]. In addition, at the end of each level, an interactive quiz is administered to ensure knowledge retention. Game incentives are provided as game levels and quizzes are completed successfully.

The mechanisms of behavior change for this app were derived from social cognitive theory [39]. The social cognitive theory constructs of social modeling (game characters), mastery experience (in-app practice of IYCF recommendations), verbal persuasion (voice-acted narratives), reinforcement (rewards), facilitation (in-game modeling of support by health personnel and influential family members), and emotional state improvement (relaxing game) were used to influence dynamic and reciprocal relationships between IYCF behaviors, personal factors (knowledge, self-efficacy, and child feeding skills), and environmental factors (social support).

#### Recipe Guide

This module presents 30 nutritionally adequate recipes derived from locally available and affordable foods [40]. Mothers are prompted to prepare the meals and take photographs of their children’s meals to be stored in the app’s database for subsequent nutritional evaluation by researchers.

#### Child Health Planner

This planner includes a calendar with reminders for available health services (birth registration, well-child clinics, immunization, deworming, vitamin A supplementation, and malaria prophylaxis) that are poorly used to date. Mothers were prompted to access these services, document activities, and upload photos of paperwork on the app to evaluate compliance.

#### Child Growth Monitor

This monitor is based on algorithms from the World Health Organization Anthro software [41]. It documents measurements of the weight and height of the child taken during child welfare clinic visits. The app plots child growth patterns using age and anthropometric indicators. If needed, it recommends corrective actions, such as more frequent meals, recipes with increased nutrient density, or clinic visits.

### BCTs Used

Six groups of BCTs [42] were used in the BabyThrive app: (1) shaping knowledge, (2) natural consequences, (3) comparison of behavior, (4) repetition and substitution, (5) reward and threat, and (6) feedback and monitoring; for instance, under shaping knowledge, the BCT of instruction on how to perform the behavior was implemented as detailed steps to successful breastfeeding and complementary feeding. The depiction of BCTs relating to natural consequences includes (1) Information about health consequences (in-app description of the effects of commercial infant feeds or mixed feeding), (2) salience of consequences (the benefits of exclusive breastfeeding, including reduction of risk for postpartum bleeding as well as breast and ovarian cancers), and (3) anticipated regret (the possible effects of nonexclusive breastfeeding on the child, such as increased risks of diarrhea, infections, and future chronic illnesses). A BCT relating to comparison of behaviors was demonstration of the behavior, implemented in the app via animated demonstrations of breastfeeding attachment and positions as well as breast milk expression. Behavioral practice and rehearsal (in-game interactive quizzes) and graded tasks (graded quiz responses that must be repeated until mastery is achieved) were 2 BCTs used under repetition and substitution. Reward and threat (outcome rewards) were operationalized by earning points for correct quiz responses. The BCTs concerning feedback and monitoring were self-monitoring (in-app documentation of the child’s anthropometric measurements by the mother) and feedback on the outcome or outcomes of behavior (in-app visual feedback related to the child’s anthropometric measurements and suggestions for corrective actions).

### App Validation

Previous studies have indicated that evaluations of mobile apps should encompass an analysis of their content, an assessment of usability and user satisfaction, and the testing of efficacy to assess whether there is a reliable relationship between the effects of the app and outcomes from other methods [43-46]. Content evaluation was conducted during the app development phase by nutrition experts. App usability and user satisfaction were assessed by a different group of 90 teenage mothers (aged 14-19 y) using the mHealth App Usability Questionnaire [47]. These mothers had at least 1 child aged <2 years, and they were recruited from rural communities (Sabon-Lugbe, Karmo, Angwan Sayawa, Kagini, Gosa, Iddo Pada, Dape, Kubwa, and Kabusa) in the suburbs of Abuja. The study was publicized in the target area through village heads and local information channels. Potential participants were assessed for eligibility. Eligible individuals were informed about the research procedures, risks, benefits, and freedom to opt out of the study at any time and were enrolled after they provided informed consent. The mHealth App Usability Questionnaire consists of 18 usability statements rated on a 7-point Likert scale from 1=*very strongly disagree* to 7=*very strongly agree*, with a total possible score of 7 per statement. Very few study participants (0/90, 0%-3/90, 3%) chose the *very strongly disagree* response; thus, this option was removed during analysis, and responses under this option were merged with the *strongly disagree* category. The final version of the BabyThrive app was validated by crossover design in the teenage mothers, who were required to have access to a smartphone. The crossover design was used to achieve a high statistical power, minimize the effect of interparticipant variability, and facilitate a comparison of within-participant effects (because each study participant serves as their own control) [48]. The efficacy of the app in disseminating IYCF knowledge was assessed by IYCF knowledge scores obtained from interviewer administration of the validated Teen Moms Child Feeding Questionnaire for Sub-Saharan Africa [49]. Only the knowledge scale was used; we did not use the attitude scale because the time period was considered too short to be able to observe notable changes in attitude [50,51]. The knowledge scale contains 23 questions under 3 subscales (food type, exclusive breastfeeding, and complementary feeding). The IYCF knowledge of the mothers was tested before the training, and then the 90 teenage mothers were randomly assigned by drawing lots into 2 groups with a 1:1 allocation ratio (verbal IYCF training group: n=45, 50%; BabyThrive group: n=45, 50%). Over the course of 2 days, 1 group participated in a 4-hour session of verbal IYCF training, with content adapted from the UNICEF IYCF training package for Nigeria. Simultaneously, the app was installed by research assistants on the mobile phones of participants in the second group. These respondents were trained briefly on the use of the BabyThrive app and asked to use it for 7 days due to the possibility of power failures. Each participant in both groups then received the same assessment questionnaire. After a washout period of 1 week [52,53], participants were crossed over to alternate groups to receive either the verbal IYCF training or the app. The assessment questionnaire was then administered a third time to each participant. Each participant remained in the study for approximately 3 weeks. App efficacy was determined by comparing IYCF knowledge before the training and after verbal training and BabyThrive app use. Construct validity [54,55] was established for the BabyThrive app by examining the relationship of total IYCF knowledge scores of the teenage mothers obtained after verbal training and BabyThrive app use.

### Statistical Analyses

Statistical analysis was performed using R software (version 4.2.2; R Foundation for Statistical Computing) and the RStudio integrated development environment (version 2022.07.2+554; Posit Software, PBC) [56]. Content validity was established by a panel of 10 nutrition experts. App quality rating by experts was analyzed via descriptive statistics (mean and quartiles) of Likert responses to the MARS. App usability by the teenage mothers was described by the frequencies of Likert responses to the mHealth App Usability Questionnaire. App efficacy was assessed by (1) the proportions of correct responses to the IYCF knowledge questions, with differences between the groups determined by a chi-square test; (2) mean IYCF knowledge scores for questionnaire subscales, with mean differences between the training groups determined by 1-way ANOVA and post hoc analysis by the Tukey highest significant difference test; and (3) differences in the effects of the training groups, explored using generalized estimating equations. Generalized estimating equations are an extension of the standard linear regression that takes correlations between repeated measures into consideration in analyzing longitudinal or clustered data [57,58]. Carryover effects were tested by analyzing differences in the effects of the order of group participation. Construct validity was established through Spearman correlation of the total knowledge scores between the verbal training and BabyThrive groups, with a coefficient ranging from 0.40 to 0.80 considered appropriate [54,55], and by a graphical illustration of the agreement between both groups via a Bland-Altman plot. A Bland-Altman plot visually represents the relationship between paired variables on the same measurement scale [59]. The Bland-Altman statistics were computed from the mean differences in knowledge scores as a function of the mean knowledge score from both groups. Three outlying data points were excluded from this analysis. To further explore the agreement between the verbal training and BabyThrive app groups, the mean absolute difference was computed from the differences in knowledge scores between both groups. Statistical significance for all tests was set at *P*<.05.

### Ethical Considerations

Ethics approval for the study was obtained from the institutional review board of the University of Texas at Austin (STUDY00001047) and the Federal Capital Territory Health Research Ethics Committee in Abuja (FHREC/2021/01/148/14-12-21). All participants provided informed consent before enrollment into the study, and consent was obtained from the parents or husbands of the teenage mothers in the form of a signature on the informed consent form. Each respondent received an incentive equivalent to US $5 on completing the final questionnaire. All data were deidentified, coded as participant numbers on the data sheets and questionnaires, and stored in a locked cabinet in a locked office for confidentiality purposes.

## Results

### Sociodemographic Characteristics

**Figure 2:**
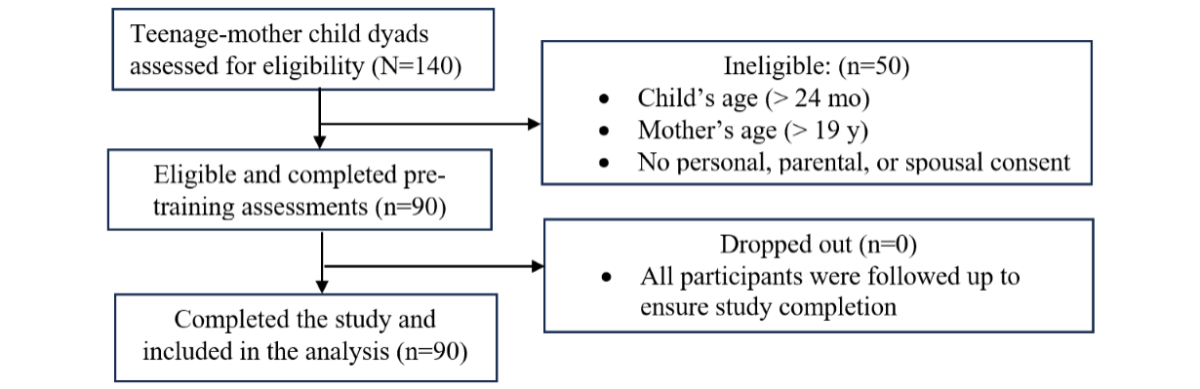
CONSORT (Consolidated Standards of Reporting Trials) flow diagram for the development and validation of the BabyThrive app.

Table 1 shows the sociodemographic characteristics of the 90 teenage mothers who participated in the validation study. Figure 2 shows the CONSORT (Consolidated Standards of Reporting Trials) flow diagram for the study. A large proportion (74/90, 82%) had attended secondary school, were married (53/90, 59%), were not working (63/90, 70%), and had no personal income (63/90, 70%). More than half of the children (49/90, 54%) were boys, and a little more than half were aged >6 months (47/90, 52%). First-born children made up the majority (74/90, 82%) of the participants.

**Table 1:** Sociodemographic characteristics of the teenage mother-child dyad from rural areas of northern Nigeria who participated in the validation study (n=90).

Characteristics			Participants, n (%)
**Mother**			
	**Age (y)**		
		15-17	6 (7)
		18-19	84 (93)
	**Education**		
		None	2 (2)
		Primary	11 (12)
		Junior secondary	30 (33)
		Senior secondary	44 (49)
		Tertiary	3 (3)
	**Marital status**		
		Single, separated, or cohabiting	37 (41)
		Married	53 (59)
	**Living with**		
		Parents, relatives, or friends	54 (60)
		Husband	36 (40)
	**Occupation**		
		None	63 (70)
		Services (eg, hairdressing, tailoring)	18 (20)
		Other (eg, petty trading, farming, teaching)	9 (10)
	**Personal monthly income, naira (US $)**		
		None	63 (70)
		≤20,000 (≤44)	25 (28)
		>20,000 (>44)	2 (2)
	**Household monthly income, naira (US $)**		
		Not known	34 (38)
		≤20,000 (≤44)	11 (12)
		>20,000 (>44)	45 (50)
**Child**			
	**Sex**		
		Male	49 (54)
		Female	41 (46)
	**Age (mo)**		
		0-5	43 (48)
		6-11	17 (19)
		12-17	15 (17)
		18-23	15 (17)
	**Birth order**		
		First	74 (82)
		Second	12 (13)
		Third	4 (4)

**Figure 3:**
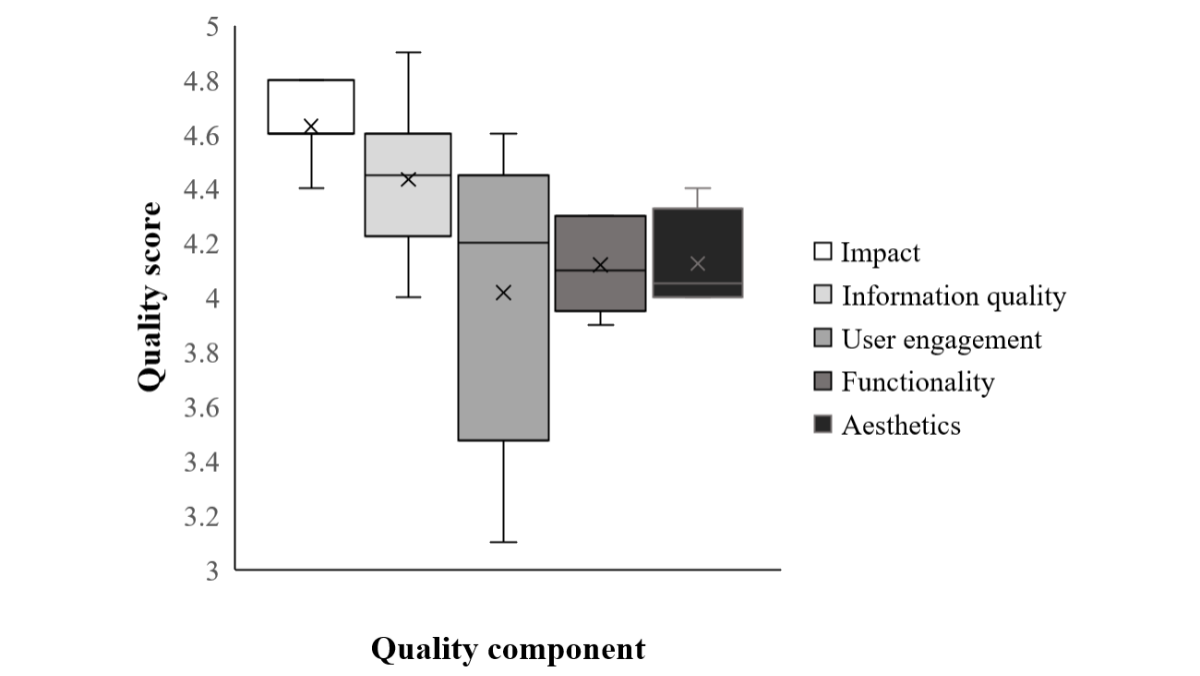
Quality ratings of the BabyThrive app given by experts using the Mobile App Rating Scale.

Figure 3 depicts the quality ratings of the BabyThrive app given by experts using the MARS. The highest mean score of 4.6 (SD 0.14) was for perceived app impact, which encompasses the ability of the app to create awareness, disseminate knowledge, influence attitudes and intention, and promote help seeking and behavior change. The mean rating for quality of app information (accuracy of description of the app and its goals as well as quality and quantity of visual and other information) was 4.4 (SD 0.29). User engagement ratings (entertainment value, ability to sustain interest in the target group, ease of customization, and interactivity) ranged widely (from 3.1 to 4.6), with a mean score of 4.0 (SD 0.58). Similar mean scores of 4.1 (SD 0.18) and 4.1 (SD 0.19) were reported for functionality ratings (gestural design, ease of use, navigation, and app performance) and aesthetic value (visual appeal, app attractiveness, and graphical quality). Overall, the mean quality rating for the BabyThrive app was 4.3 (SD 0.39).

**Figure 4:**
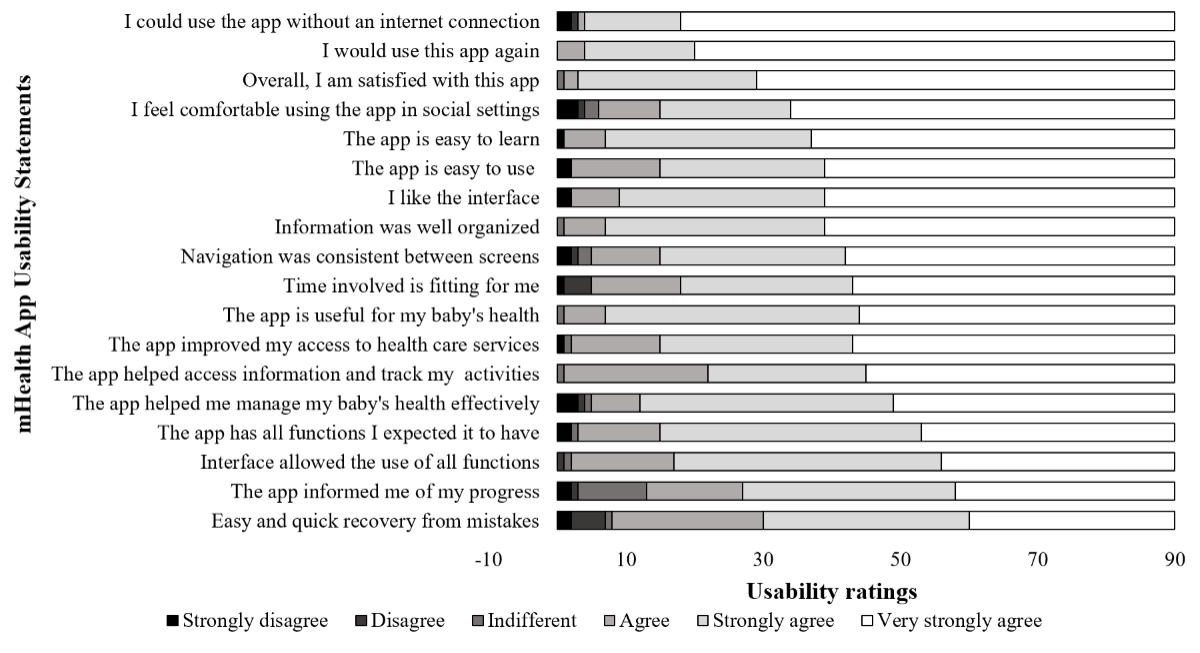
Usability ratings of the BabyThrive app given by teenage mothers using the mHealth App Usability Questionnaire.

Figure 4 displays the usability ratings of the BabyThrive app given by teenage mothers using the mHealth App Usability Questionnaire. Very few teenage mothers strongly disagreed (1/90, 1%-3/90, 3%), disagreed (1/90, 1%-5/90, 6%), or were indifferent (1/90, 1%-10/90, 11%) to the usability statements. Rather, the majority of the participants (77/90, 86%-90/90, 100%) expressed some measure of agreement. A large proportion of participants very strongly agreed that they could use the BabyThrive app without an internet connection (72/90, 80%), would use the app again (70/90, 78%), and were satisfied with the app (61/90, 68%). Fewer participants very strongly agreed that they could easily and quickly recover from mistakes (30/90, 33%), the app informed them of their progress (32/90, 36%), and the interface allowed them to use all app functions (34/90, 38%).

### Child Feeding Knowledge

Tables 2 and 3 summarize the child feeding knowledge of the teenage mothers by crossover design before the training and after verbal training and BabyThrive app use. Chi-square tests showed that IYCF knowledge of the teenage mothers (characterized by correct responses to the knowledge questions) was significantly higher in those who received either the verbal training or the BabyThrive app first compared with those before receiving any training. Before the training, knowledge concerning how a mother can keep up her breast milk supply (8/90, 9%), how long breast milk can keep without refrigeration (14/90, 16%), and reasons for allowing the baby to suckle on 1 breast (before changing to the other) for a specific length of time (23/90, 26%) were the lowest. The highest proportions of correct responses before the training were reported for knowledge concerning the first food appropriate for a newborn (74/90, 82%); how often an infant aged <6 months should be breastfed daily (67/90, 74%); and actions to take when an infant is vomiting, stooling, or convulsing (66/90, 73%).

**Figure 5:**
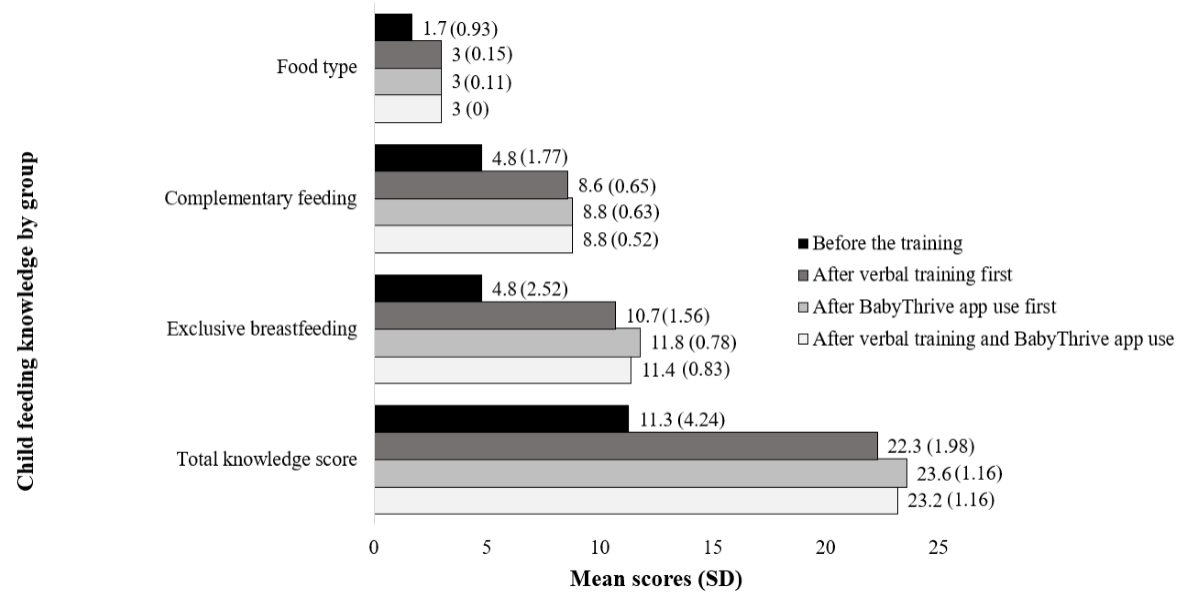
Mean (SD) scores for child feeding knowledge before the training and after verbal training and BabyThrive app use.

Furthermore, there were no significant differences in knowledge between those who started with either the verbal training or the BabyThrive app, except in the case of 6 questions. Those who started with the app had significantly higher proportions of correct responses. The 6 questions included knowledge concerning reasons why infants should receive either thick or watery porridges (39/45, 98% vs 44/45, 87%; *P*=.04), how a mother can maintain her breast milk supply (25/45, 78% vs 35/45, 56%; *P*<.001), the benefits of exclusive breastfeeding for mothers (34/45, 100% vs 45/45, 76%; *P*=.001), actions to overcome difficulties with breastfeeding (41/45, 100% vs 45/45, 91%; *P*=.04), how long and why an infant should be allowed to suckle on 1 breast before changing to the other (45/45, 100% vs 36/45, 80%; *P*=.002; and 45/45, 100% vs 28/45, 62%; *P*=.02, respectively), and signs that an infant is getting enough milk (43/45, 96% vs 31/45, 69%; *P*<.001). Figure 5 presents mean differences in IYCF knowledge scores across the training groups. All pretraining mean knowledge scores were significantly lower than the scores in both the verbal training and BabyThrive groups as well as after verbal training and BabyThrive app use. Knowledge of food type and complementary feeding did not differ significantly. Knowledge of exclusive breastfeeding (*P*<.001) and total knowledge scores (*P*=.002) were significantly higher in the BabyThrive group than in the verbal training group.

**Table 2:** Correct responses of child feeding knowledge by crossover design before the training and after verbal training and BabyThrive mobile app use.

		Correct responses	
		Before the training^a^ (n=90), n (%)	After verbal training and BabyThrive mobile app use^b^ (n=90), n (%)
**Food type**			
	What is the first food for a newborn?	74 (82)	90 (100)
	What should infants <6 months be fed?	44 (49)	90 (100)
	When a mother resumes work, what should her infant be fed?	34 (38)	90 (100)
**Complementary feeding**			
	At what age should complementary foods be introduced?	62 (69)	85 (94)
	Why should complementary foods be introduced at the stated age?	61 (68)	90 (100)
	How many times daily should a 6-month-old receive complementary foods?	43 (48)	90 (100)
	What quantity of food should an 8-month-old receive per meal?	42 (47)	88 (98)
	Should an infant receive thick or watery pap or porridges? Give reasons.	50 (56)	85 (94)
	What should a mother do when her infant is vomiting, stooling, and convulsing?	66 (73)	90 (100)
	How should you feed an infant who is refusing to eat complementary food?	29 (32)	90 (100)
	What kind of diet provides the greatest amount of nutrients?	29 (32)	89 (99)
**Exclusive breastfeeding**			
	How long should infants be fed with only breast milk after birth?	58 (64)	90 (100)
	Why should infants be fed with breastmilk alone for some time after birth?	54 (60)	90 (100)
	How often should an infant <6 months be breastfed daily?	67 (74)	90 (100)
	How can a mother keep up her breast milk supply?	8 (9)	78 (87)
	What are the benefits of feeding an infant with breast milk only for mothers?	18 (20)	84 (93)
	How long can expressed breast milk keep without refrigeration?	14 (16)	89 (99)
	What should a mother do to overcome difficulties with breastfeeding?	27 (30.0)	90 (100)
	What are the effects of commercial or mixed feeding in infants <6 months?	57 (63)	90 (100)
	What are the signs that an infant needs to be breastfed?	59 (66)	88 (98)
	How long should an infant be suckled on 1 breast?	24 (27)	89 (99)
	Why should an infant suckle on 1 breast for the stated length of time?	23 (26)	83 (92)
	What are the signs that an infant is getting enough breast milk?	25 (28)	88 (98)

^a^All pretraining proportions of correct responses were significantly lower than in both the verbal training and BabyThrive groups (*P*<.05).

^b^All postverbal training and post–BabyThrive app use proportions of correct responses were significantly higher than pretraining proportions (*P*<.05).

**Table 3:** Correct responses of child feeding knowledge by crossover design in participants who received verbal training initially and the BabyThrive mobile app initially (n=90).

		Correct responses	
		After receiving verbal training initially (n=45), n (%)	After receiving the BabyThrive mobile app initially (n=45), n (%)
**Food type**			
	What is the first food for a newborn?	45 (100)	45 (100)
	What should infants <6 months be fed?	43 (96)	44 (98)
	When a mother resumes work, what should her infant be fed?	45 (100)	45 (100)
**Complementary feeding**			
	At what age should complementary foods be introduced?	41 (91)	41 (91)
	Why should complementary foods be introduced at the stated age?	44 (98)	45 (100)
	How many times daily should a 6-month-old receive complementary foods?	45 (100)	45 (100)
	What quantity of food should an 8-month-old receive per meal?	43 (96)	43 (96)
	Should an infant receive thick or watery pap or porridges? Give reasons.	39 (87)	44 (98)^a^
	What should a mother do when her infant is vomiting, stooling, and convulsing?	45 (100)	45 (100)
	How should you feed an infant who is refusing to eat complementary food?	44 (98)	45 (100)
	What kind of diet provides the greatest amount of nutrients?	40 (89)	43 (96)
**Exclusive breastfeeding**			
	How long should infants be fed with only breast milk after birth?	44 (98)	45 (100)
	Why should infants be fed with breastmilk alone for some time after birth?	43 (96)	45 (100)
	How often should an infant <6 months be breastfed daily?	45 (98)	45 (100)
	How can a mother keep up her breast milk supply?	25 (56)	35 (78)^a^
	What are the benefits of feeding an infant with breast milk only for mothers?	34 (76)	45 (100)^a^
	How long can expressed breast milk keep without refrigeration?	43 (96)	45 (100)
	What should a mother do to overcome difficulties with breastfeeding?	41 (91)	45 (100)^a^
	What are the effects of commercial or mixed feeding in infants <6 months?	44 (98)	45 (100)
	What are the signs that an infant needs to be breastfed?	43 (96)	45 (100)
	How long should an infant be suckled on 1 breast?	36 (80)	45 (100)^a^
	Why should an infant suckle on 1 breast for the stated length of time?	28 (62)	45 (100)^a^
	What are the signs that an infant is getting enough breast milk?	31 (69)	43 (96)^a^

^a^Proportions of correct responses were significantly higher in the BabyThrive group than in the verbal training group (*P*<.05).

### A Comparison of Child Feeding Knowledge

Table 4 presents a comparison of child feeding knowledge using generalized estimating equations. Knowledge of food type was approximately 1.3 times higher after verbal training and BabyThrive app use than before the training (*P*<.001). Similarly, both the verbal training and BabyThrive groups had significantly increased (>3-fold) knowledge concerning complementary feeding in comparison with before the training (*P*<.001). Knowledge of exclusive breastfeeding was approximately 5.91 times greater after verbal training and 6.99 times higher after BabyThrive app use than before the training (*P*<.001). Total knowledge scores after verbal training and BabyThrive app use exceeded pretraining scores by approximately 11-fold and 12-fold, respectively (*P*<.001). In the BabyThrive group, exclusive breastfeeding knowledge (odds ratio [OR] 1.08, 95% CI 0.65 to 1.50) and total knowledge scores (OR 1.29, 95% CI 0.72 to 1.86) were significantly greater than in the verbal training group (*P*<.001). No differences were observed between the verbal training and BabyThrive groups with respect to knowledge of food type (*P*=.83) and complementary feeding (*P*=.63). No carryover effects were recorded because the order of group participation had no significant effects on the outcomes of the training. Knowledge of food type did not differ in participants who received the verbal training first compared with those who received the BabyThrive app first and vice versa (OR 0.033, 95% CI –0.056 to 0.034; *P*=.18). Similarly, knowledge of complementary feeding (OR 0.03, 95% CI –0.29 to 0.22; *P*=.95), exclusive breastfeeding (OR 0.31 95% CI –0.02 to 0.94; *P*=.24), and total knowledge scores (OR 0.44; 95% CI –0.17 to 1.06, *P*=.21), did not differ significantly by order of treatment.

**Figure 6:**
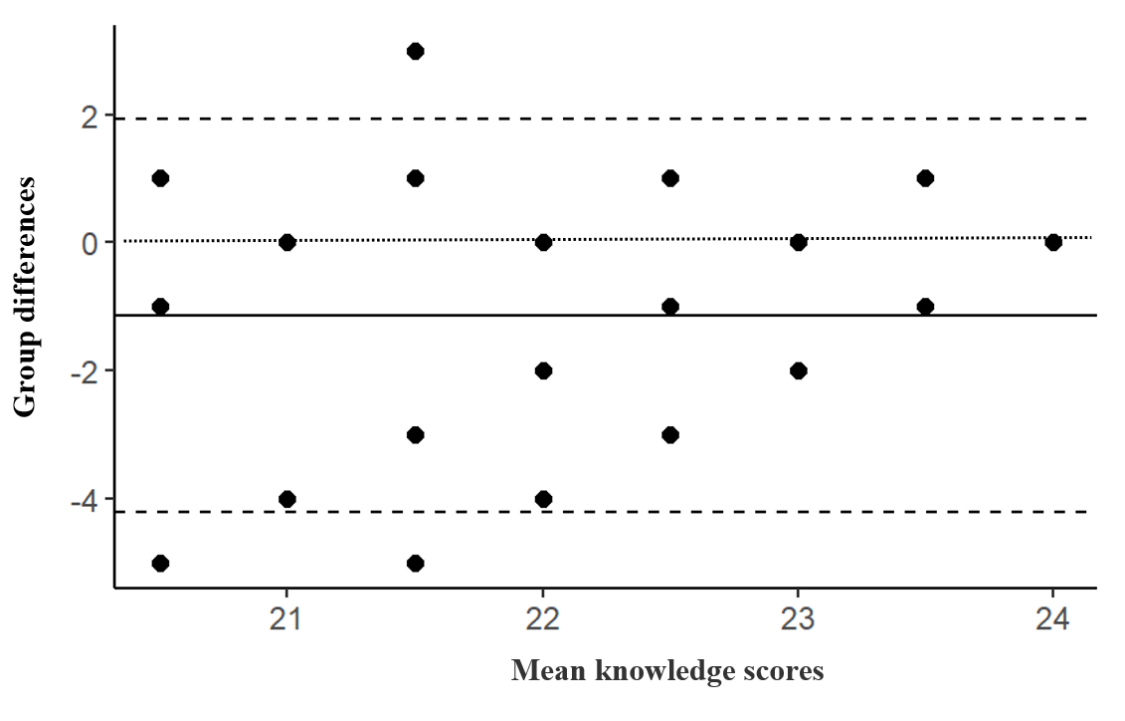
Bland-Altman plot depicting agreement in child feeding knowledge scores after verbal training and BabyThrive app use.

Figure 6 presents a Bland-Altman plot depicting agreement in IYCF knowledge scores between the verbal training and BabyThrive groups. The vertical axis represents the mean differences in IYCF knowledge scores, while the horizontal axis represents the mean IYCF knowledge scores of the verbal training and the BabyThrive groups. The dotted line at the 0 point on the vertical axis indicates an ideal situation where there is zero difference in IYCF knowledge scores from the verbal training and the BabyThrive app. The solid line represents the mean absolute difference in child feeding knowledge between the verbal training and BabyThrive groups. The dashed line above the solid line denotes the upper limit of agreement between both methods, while the lower dashed line shows the lower limit of agreement. Both methods can be said to be in agreement because 80% of the plot points lie within the limits of agreement. Thus, the BabyThrive app is a more effective tool to train teenage mothers on child feeding in place of the verbal IYCF training. The mean absolute difference in knowledge scores between the verbal training and BabyThrive groups was –1.14, with upper and lower limits of agreement of 1.93 and –4.21, respectively. The IYCF knowledge scores obtained from both groups showed coherence, with a statistically significant Spearman correlation coefficient of 0.50 (*P*<.001).

**Table 4:** Comparison of child feeding knowledge using generalized estimating equations.

Child feeding knowledge across groups		β estimate (regression coefficient, SE; 95% CI)	Z statistic	*P* value
**Food type**				
	Before the training	Reference	Reference	Reference
	Verbal training group	1.29 (0.01; 1.06 to 1.52)	12.95	<.001^a^
	BabyThrive group	1.30 (0.01; 1.07 to 1.53)	13.14	<.001^a^
	BabyThrive group vs verbal training group	0.01 (0.02; –0.06 to 0.03)	0.58	.83
**Complementary feeding**				
	Before the training	Reference	Reference	Reference
	Verbal training group	3.43 (0.23; 2.85 to 3.95)	14.56	<.001^a^
	BabyThrive group	3.47 (0.23; 2.95 to 4.05)	14.93	<.001^a^
	BabyThrive group vs verbal training group	0.10 (0.11; –0.15 to 0.35)	0.92	.63
**Exclusive breastfeeding**				
	Before the training	Reference	Reference	Reference
	Verbal training group	5.91 (0.32; 5.17 to 6.65)	18.68	<.001^a^
	BabyThrive group	6.99 (0.27; 6.36 to 7.62)	26.00	<.001^a^
	BabyThrive group vs verbal training group	1.08 (0.18; 0.65 to 1.50)	5.97	<.001^a^
**Total knowledge score**				
	Before the training	Reference	Reference	Reference
	Verbal training group	11.03 (0.50; 9.86 to 12.21)	21.95	<.001^a^
	BabyThrive group	12.32 (0.46; 11.24 to 13.40)	26.81	<.001^a^
	BabyThrive group vs verbal training group	1.29 (0.24; 0.72 to 1.86)	5.29	<.001^a^

^a^Significant *P* value.

## Discussion

### Principal Findings

This study developed and validated the BabyThrive app, a mobile gaming app aimed at improving the knowledge of IYCF practices among teenage mothers in Nigeria. The app is an easy, low-cost method to distribute educational content about child nutrition to mothers in rural communities in Nigeria. This interactive mobile app combines active engagement with learning critical nutritional information. Electronic media–based interventions, such as mobile apps and video games, offer distinct advantages over conventional methods of health education because they may be less expensive and more effective in producing behavior change [27,28]. The BabyThrive app distinguishes itself from other apps by focusing on IYCF practices to diminish malnutrition in very young children. It uses gaming technology, interactive videos, and audio techniques adapted to a local Nigerian language as a means of information delivery.

BabyThrive was created using a stepwise process based on existing evidence and resources, as well as qualitative and quantitative inputs and assessments by target users and experts. Quality ratings given by experts (≥4.0 out of 5) and usability ratings given by teenage mothers (>80% in agreement with usability statements) were high. The BabyThrive app improved maternal IYCF knowledge 12-fold in comparison with baseline status. It performed 1.29 times better than the verbal training in transmitting IYCF knowledge to teenage mothers in Nigeria. The IYCF scores from the verbal training were slightly lower but in agreement with scores from the app, as reflected in a small mean absolute difference of 1.14 (which is negligible on the 23-point knowledge scale). Similarly, IYCF knowledge scores obtained from both training methods showed coherence, with a statistically significant, moderate correlation of 0.50. These data suggest that the BabyThrive app is equivalent to, and even surpasses, the verbal training in disseminating IYCF knowledge.

The current research found high levels of user acceptability, but in an evaluation of the use of a breastfeeding tracking app in the United States, user experiences were mixed (helpful, excessive time required, and anxiety inducing) [60]. The high levels (>80%) of usability and user satisfaction in our study are consistent with the findings of >80% satisfaction with the Growing Healthy mHealth app in Australia [61]. In Australian mothers, similarly high levels (>80%) of user agreement with usability statements were reported concerning the Milky Way app for breastfeeding support [62]. In rural India, the breastfeeding support app, Best4Baby, reported high usability values (>95th percentile) [63]. On the basis of assessments conducted using the mHealth App Usability Questionnaire (which was also used in our study), high usability and user satisfaction values were reported for the BabyByte app, which was designed to promote responsive feeding practices in parents of infants and toddlers in the United States [64]. In Malaysia, a wide range of positive responses to usability statements (50%-100%) was reported for the Gigiku Sihat app for caregiver training on child diet and oral health. These results differ from those of our study, which had a narrower range (85%-100%) of agreement with usability statements [65]. In comparison with the values (100% for both ease of use and ease of learning the app) reported for the Gigiku Sihat app [65], similarly high proportions of participants (97.8% and 98.8%, respectively) in our study had positive responses to statements concerning ease of use and ease of learning the app. In Egypt, an overall mean app quality rating of 3.7 was reported for the Sehhat Tefly app, which was aimed at improving the capacity of caregivers to track the health and well-being of children [66]. By contrast, using the same quality assessment scale as the Sehhat Tefly app [66], our study had a greater overall mean app quality rating of 4.3 (SD 0.39).

Among mothers living in rural Nepal, the serious game MANTRA, which used simple game mechanics such as “drag and drop,” recorded an average gain of 6.0 points (on a 100-point scale) in knowledge concerning neonatal health [30]. This mechanism was also used in our BabyThrive app. In comparison with mHealth studies in Pakistan (14.8% increase) and Australia (6.9% increase), our BabyThrive app successfully increased IYCF knowledge of mothers to a much higher degree (12-fold over pretraining levels) [67,68]. In Ethiopia, an mHealth study to promote exclusive breastfeeding knowledge in 121 parent pairs recorded a lower mean difference in IYCF knowledge (>7.0) than that in our investigation (12.3) [69]. Similarly, the knowledge of 720 Sri Lankan mothers concerning child feeding also increased significantly in an mHealth intervention [70].

Numerous studies on mHealth apps aimed at improving maternal knowledge of IYCF practices have compared app efficacy to either baseline or controls with no treatment [30,67-69]. Only a few mHealth studies have compared app efficacy to other methods for achieving the same outcomes. Our study compared app efficacy to baseline (before the training), as well as to outcomes of verbal training (the usual method of IYCF knowledge dissemination). Despite the mixed results on user experience shown on the breastfeeding tracking app in the United States [60], the agreement (intraclass correlation coefficient of 0.97) with the interview method was notably higher than the moderate Spearman correlation coefficient of 0.50 documented in our study. Our lower correlation may be because the app was 1.29-fold more effective than the verbal training in transmitting IYCF knowledge. Similar to our findings, the use of the Khunlook mobile app given to parents in Thailand greatly increased health literacy in comparison to an intervention delivered via a child health handbook [71], and consistent with our findings, a complementary feeding education trial in Iran reported an 18-point difference in maternal nutrition literacy between mHealth app users and controls who received regular well-child services [72].

We used a mobile game as the vehicle for change because gamification has been shown to have great potential for improving knowledge and changing behavior through reinforcement and personalized or tailored approaches [73-77]. Games appeal to broad audiences, can trigger motivation to initiate and sustain desired behaviors, and promote well-being by inducing positive emotions and experiences [74-79]. In particular, mobile games are suitable for health interventions in adolescents, given their high proclivity for playing games and using technology [76,79].

The BabyThrive app was created with the purpose of training teenage mothers considered to be at risk with malnourished infants to gain knowledge and learn practices to improve their child’s nutritional health. We believe that this improvement has the potential to reduce child morbidity and mortality, lower health care costs for families, and improve intergenerational family income and overall prosperity of the community. This app intervention will be sustainable because the app can be widely disseminated through web-based app stores, development partners, health facilities, and community volunteers; in addition, app maintenance (periodic updates and app store fees) involves minimal costs. The UNICEF IYCF training package (on which the BabyThrive app is based) is widely used in sub-Saharan Africa and in >90 countries globally [80]. The results of this study can be generalized to adolescent mothers in rural areas of Nigeria and in countries where this training package is used; however, the specific findings may vary across different settings. Furthermore, as the BabyThive app is available in 2 languages, templates exist for the translation of the app into other languages. Thus, with cultural adaptation of the recipes contained in the app to fit local contexts, the BabyThrive app can be easily adapted for use in other countries in sub-Saharan Africa as well as low- and middle-income nations.

### Strengths and Limitations

The strengths of this validated app include the use of gaming technologies that promote user engagement, ease of use despite unreliable internet connectivity due to offline functionality, availability in >1 language, and ease and acceptability of use among teenage mothers with low-income status in rural Nigeria. A major strength of this research is the inclusion of populations of teenage mothers who are commonly excluded from technology-driven solutions due to low household income, poor literacy levels, and language barriers. One limitation of this study is the absence of social peer-to-peer connection due to the offline nature of the app. Another limitation is that a large proportion of the participants (84/90, 93%) who participated in the validation study were aged >17 years because very few younger mothers (6/90, 7%) met the eligibility criteria.

### Conclusions

This research developed a novel, offline mobile gaming app. This app has been documented to be a valid, easy, effective, and acceptable method to disseminate critical knowledge on IYCF practices to teenage mothers in rural Nigeria. This app will be a useful instrument for professionals and personnel in the nutrition, community health, governmental, nonprofit, and child health fields, as well as other stakeholders, to improve maternal awareness of IYCF practices. Randomized controlled trials should be conducted to test the effectiveness of the BabyThrive app in improving maternal IYCF knowledge, attitudes, and practices. In addition, the ability of the app to ameliorate macro- and micronutrient undernutrition in childhood, particularly among children born to teenage mothers, should be evaluated.

## Appendix

Multimedia Appendix 1 Screenshots of the BabyThrive app.

## Abbreviations

BCT: behavior change technique
CONSORT: Consolidated Standards of Reporting Trials
IYCF: infant and young child feeding
MARS: Mobile App Rating Scale
mHealth: mobile health
OR: odds ratio
UNICEF: United Nations Children’s Fund
