# Supplementary figures and images for: A Mobile Gaming App to Train Teenage Mothers on Appropriate Child Feeding Practices: Development and Validation Study
Source: J Med Internet Res. 2024 Sep 26;26:e53560. doi: 10.2196/53560 (PMC11467632; doi:10.2196/53560)

## Multimedia Appendix 1: Screenshots of the BabyThrive app

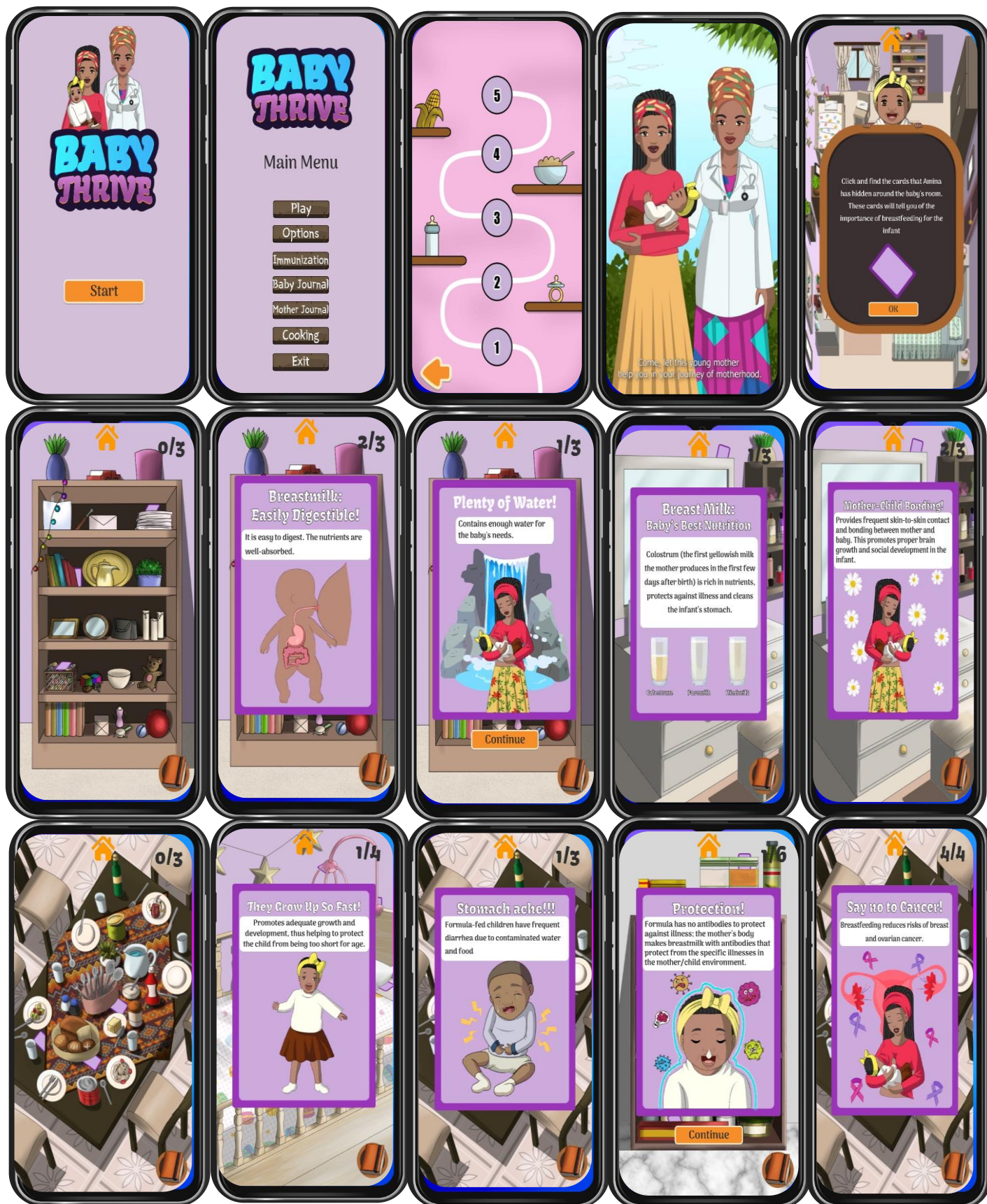

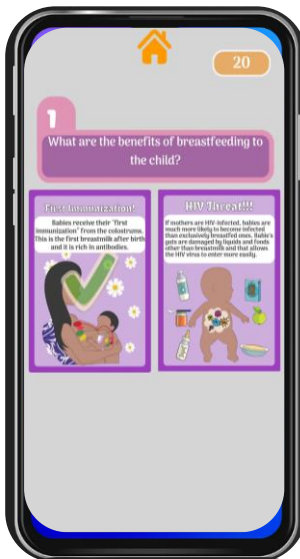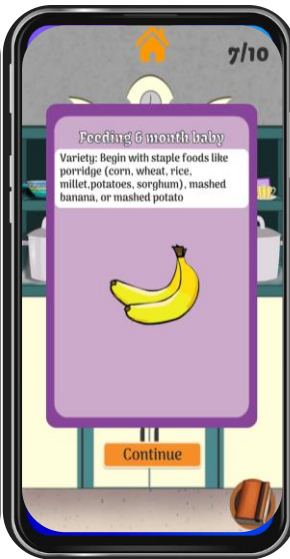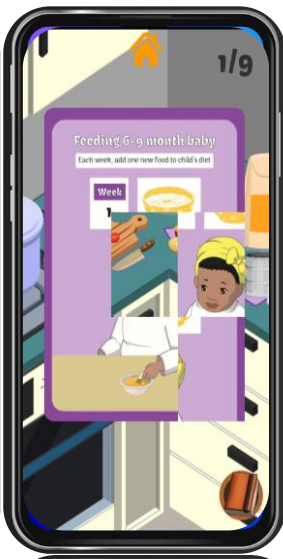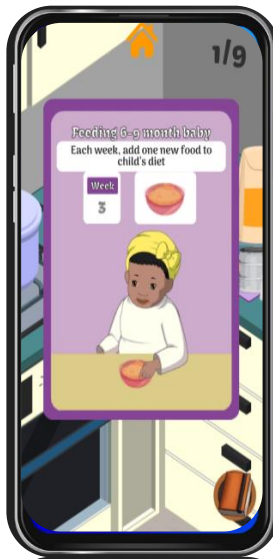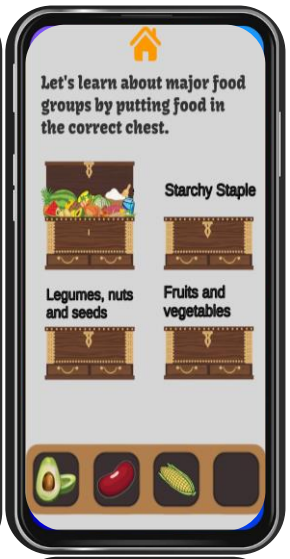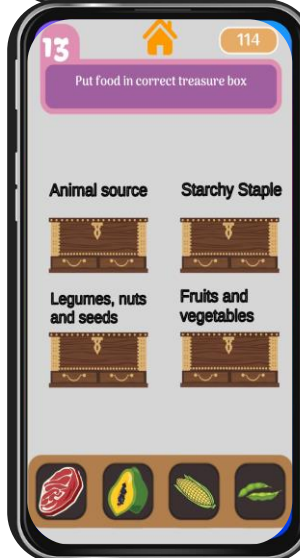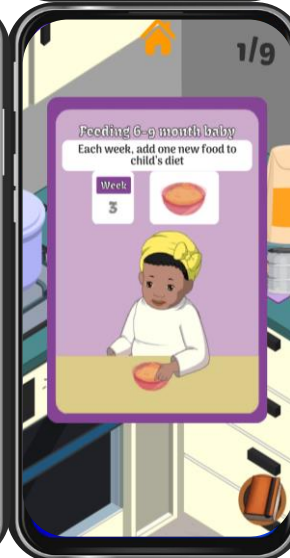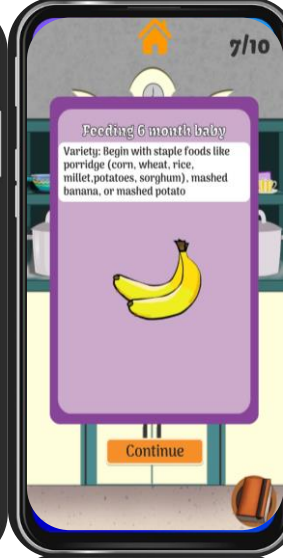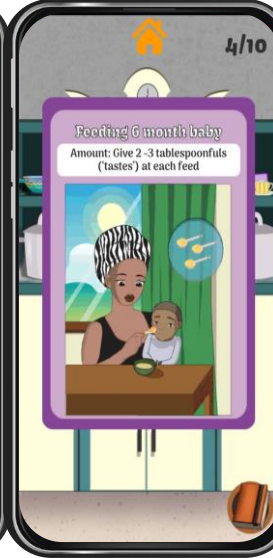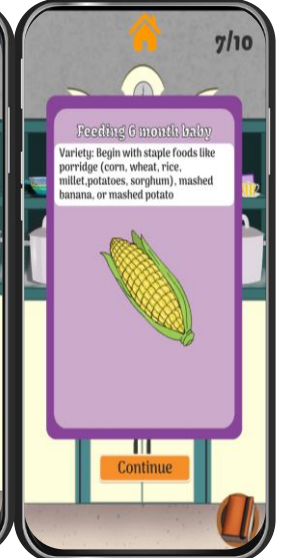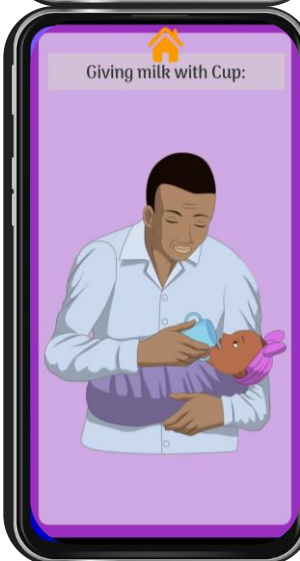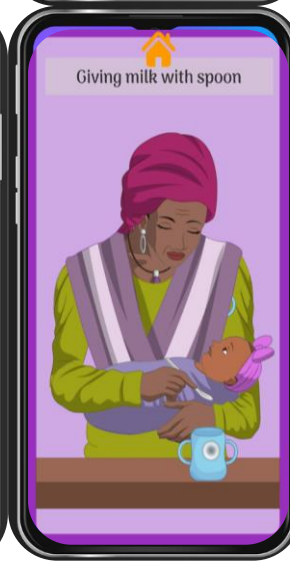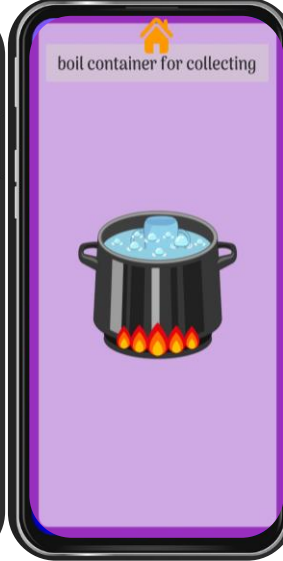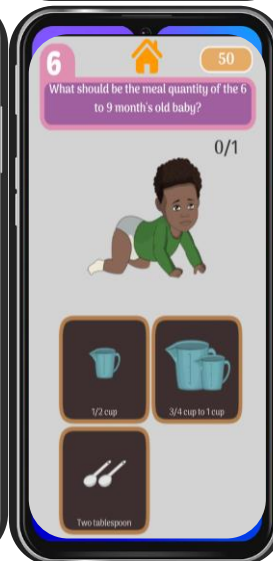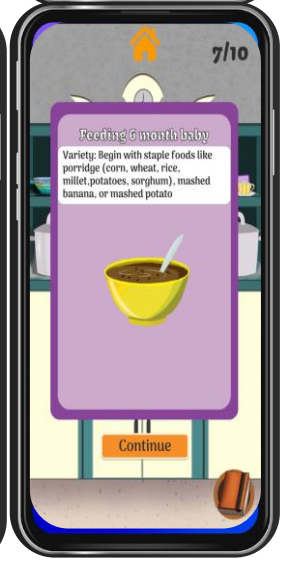

Supplement: Multimedia Appendix 1 [file jmir_v26i1e53560_app1.pdf]
